# Hormonal impact of the 17*α*-hydroxylase/C_17,20_-lyase inhibitor abiraterone acetate (CB7630) in patients with prostate cancer

**DOI:** 10.1038/sj.bjc.6601879

**Published:** 2004-05-18

**Authors:** A O'Donnell, I Judson, M Dowsett, F Raynaud, D Dearnaley, M Mason, S Harland, A Robbins, G Halbert, B Nutley, M Jarman

**Affiliations:** 1 Royal Marsden NHS Trust, Sutton, Surrey SM2 5PT, UK; 2 CR UK Centre for Cancer Therapeutics, Institute of Cancer Research, Sutton, Surrey SM2 5NG, UK; 3 Academic Department of Biochemistry, Royal Marsden Hospital, Fulham Road, London SW3 6JJ, UK; 4 Department of Clinical Oncology, Velindre Hospital, Whitchurch, Cardiff CF4 7XL, UK; 5 Department of Oncology, University College of London, The Middlesex Hospital Mortimer St, London W1N 8AA, UK; 6 Drug Development Office, Cancer Research UK, PO Box 123, London WC2A 3PX, UK; 7 Cancer Research UK Formulation Unit, University of Strathclyde, Glasgow G1 1XW, UK; 8 Institute of Cancer Research, Sutton, Surrey SM2 5NG, UK

**Keywords:** prostate cancer, hormonal therapy, pharmacokinetics, clinical study

## Abstract

A series of three dose escalating studies were conducted to investigate the ability of the 17*α*-hydroxylase/C_17,20_-lyase inhibitor abiraterone acetate, to cause maximum suppression of testosterone synthesis when delivered to castrate and noncastrate males with prostate cancer. Study A was a single dose study in castrate males. Study B was a single dose study in noncastrate males and study C was a multiple dose study in noncastrate males. The drug was given orally in a once-daily dose and blood samples taken to assess pharmacokinetic (PK) parameters and hormone levels in all patients. The study drug was well tolerated with some variability in PKs. Suppression of testosterone levels to <0.14 nmol l^−1^ was seen in four out of six castrate males treated with a single dose of 500 mg. At 800 mg given days 1–12 in noncastrate males, target suppression was achieved in three out of three patients, but a two- to three-fold increase of Luteinising Hormone (LH) levels in two out of three patients overcame suppression within 3 days. All patients in the multiple dose study developed an abnormal response to a short Synacthen test by day 11, although baseline cortisol levels remained normal. This is the first report of the use of a specific 17*α*-hydroxylase/_17,20_-lyase inhibitor in humans. Repeated treatment of men with intact gonadal function with abiraterone acetate at a dose of 800 mg can successfully suppress testosterone levels to the castrate range. However, this level of suppression may not be sustained in all patients due to compensatory hypersecretion of LH. The enhanced testosterone suppression achieved in castrate men merits further clinical study as a second-line hormonal treatment for prostate cancer. Adrenocortical suppression may necessitate concomitant administration of replacement glucocorticoid.

Prostate cancer continues to present an enormous challenge in the UK, where it is the second most common cause of cancer death in men, causing over 9000 deaths per year (Cancer Research UK, 2002).

The beneficial effect of androgen ablation on metastatic prostate cancer was realised in the 1940s, when Huggins and Hodges observed an antitumour response, as measured by a reduction in serum acid phosphatase, in patients treated by surgical or medical castration (Denmeade and Isaacs, 2002). In general, androgen deprivation will give a response of varying duration in 80–90% of men with advanced disease (Denis and Murphy, 1993). Although the multistep pathway of androgen production resulting in testosterone (Figure 1

**Figure 1:**
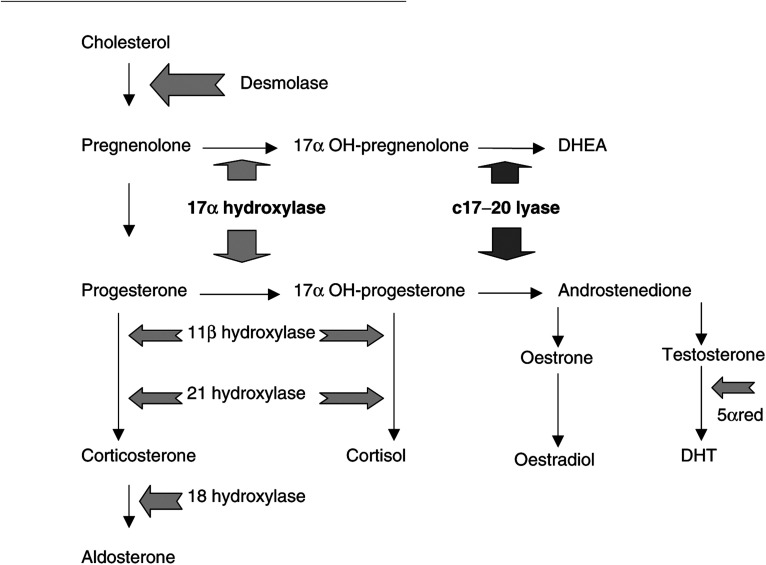
Steroid synthesis pathway. DHEA=dehydroepiandrosterone; 5*α*red=5*α*reductase; DHT=dihydrotestosterone.

) is present only in its entirety within the testes, the adrenal gland is also capable of releasing DHEA and androstenedione. Enzymes for the subsequent conversion of DHEA and androstenedione to testosterone are present in a variety of peripheral tissues as well as the prostate (Griffin and Wilson, 1998).

Studies have shown that these extratesticular sources of testosterone represent an important alternative source of androgen stimulation in a significant proportion of patients with prostate cancer. As much as 10% of baseline circulating testosterone remains in castrated men, due to peripheral conversion of adrenal steroids to testosterone (Hellerstedt and Pienta, 2002). First-line treatment of prostate cancer by androgen deprivation is generally achieved by medical or surgical testicular castration. This leaves the testosterone derived from adrenal sources intact.

It is recognised that the development of androgen-independent prostate cancer is caused, in part, by changes in androgen receptor regulation, activation of the androgen receptor being mitogenic in this malignancy. The sensitivity of the androgen receptor is increased by the overexpression of two nuclear coactivators: transcriptional intermediary factor 2 and steroid receptor coactivator 1. Transactivation of the androgen receptor is thus enhanced at lower concentrations of testosterone (Gregory *et al*, 2001a, 2001b). Furthermore, amplification of the number of androgen receptors can be shown in hormone refractory tumours, when compared to both benign prostatic hyperplasia and primary prostatic malignancy, using both reverse transcription PCR and fluorescence *in situ* hybridisation (Bubendorf *et al*, 1999; Linja *et al*, 2001).

The imidazole derivative ketoconazole and the aromatase inhibitor aminoglutethimide have been evaluated as possible agents with which to achieve decreased production of adrenal steroids. Ketoconazole is relatively unselective, inhibiting both cholesterol side chain cleavage and 11*β*-hydroxylation (Loose *et al*, 1983). A direct antitumour effect of ketoconazole *in vitro* has also been demonstrated (Eichenberger and Trachtenberg, 1988). The action of aminoglutethimide is primarily to block the formation of pregnenolone from cholesterol but it is also recognised to inhibit 11*β*-hydroxylase and peripheral aromatase (Schwimmer and Parker, 1996). In clinical trials, both agents have shown some activity as second-line agents (measured by clinical benefit as well as reduction in PSA), supporting the concept of a more selective inhibitor of the 17*α*-hydroxylase/C_17,20_-lyase enzyme (Oh, 2002).

The novel 17*α*-hydroxylase/C_17,20_-lyase inhibitor abiraterone acetate (Figure 2

**Figure 2:**
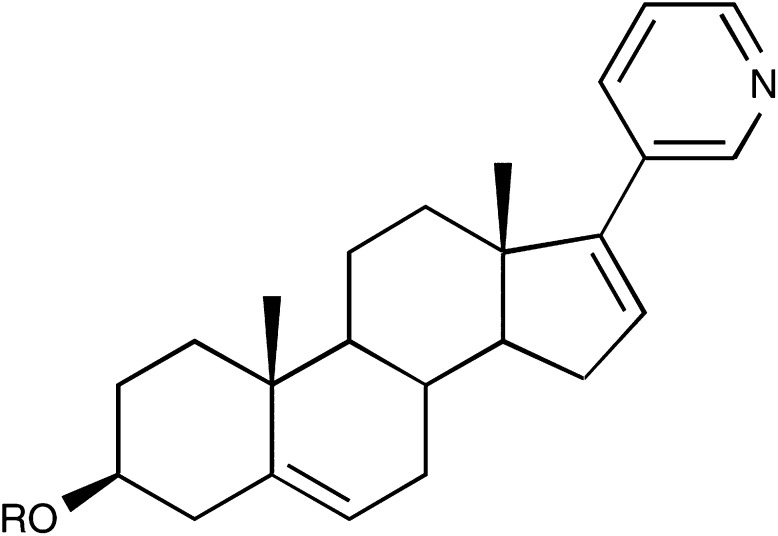
Abiraterone structure. R=H: abiraterone (CB7598); R=Ac: abiraterone acetate (CB7630).

) was developed as a mechanism-based steroidal inhibitor following observations that nonsteroidal 3-pyridyl esters had improved selectivity for inhibition (Rowlands *et al*, 1995). Abiraterone acetate is the 3-acetate and a prodrug form of CB7598 (17-(3-pyridyl)androsta-5,16-dien-3*β*-ol, abiraterone), a potent inhibitor of the enzyme with a Ki_app_ of 0.5 nM (Barrie *et al*, 1997; Potter *et al*, 1995).

Using a rodent model, following intraperitoneal administration, abiraterone acetate showed rapid deacetylation. Levels of deacetylated drug reached >1 *μ*M at 6 h, with persistence of relatively high levels of compound (approximately 0.3 *μ*M) for at least 24 h. Although this finding may be indicative of depot characteristics of the mode of administration used, it may also indicate a degree of enterohepatic recirculation that could prove favourable in the clinical setting, providing sustained target enzyme inhibition.

In these preclinical studies, there was correlative evidence of inhibition of 17*α* hydroxylation as shown, for example, by the reduced weight of the ventral prostate (Barrie *et al*, 1994; Dowsett *et al*, 1988).

Here, we describe a series of three phase one trials in which abiraterone acetate was tested in humans for the first time. This is the first report of the effects of a specific 17*α*-hydroxylase/C_17,20_-lyase inhibitor in humans. The studies were conducted to determine the dose of abiraterone acetate that will result in maximum suppression of testosterone and to obtain safety, pharmacokinetic (PK) and endocrine data, the latter to determine the specificity of inhibition. All three studies involved patients with advanced, that is, unresectable, prostate cancer.

## METHODS

Patients were recruited to one of the following three studies that were conducted in sequence. The study protocols were approved by the Research Ethics Committee of all participating institutions, and all patients gave written informed consent prior to inclusion. All three studies were conducted under the auspices of the Cancer Research UK Phase I/II Committee.

### Study A

This was a single dose study in males with castrate levels of testosterone (testosterone ⩽2 nmol l^−1^) following orchidectomy or Gonadotrophin-Releasing Hormone agonist (GnRHa) therapy, to determine the dose of abiraterone acetate that was sufficient to cause suppression of testosterone synthesis to undetectable levels (<0.14 nmol l^−1^). Significant suppression was defined as either testosterone <0.14 nmol l^−1^ in individual patients with a pretreatment value of <0.6 nmol l^−1^ or a ⩾75% reduction in individual patients with a pretreatment value of ⩾0.6 nmol l^−1^.

### Study B

This was a single dose study in noncastrate males (testosterone level ⩾9.0 nmol l^−1^) to determine the dose of abiraterone acetate that was sufficient to cause suppression of testosterone synthesis to castrate levels (⩽2.0 nmol l^−1^).

### Study C

This was a multidose study (Days 1–12) in noncastrate males to determine the dose of abiraterone acetate that was sufficient to cause persistent suppression of testosterone synthesis to castrate levels. If this level of suppression was achieved, then it was planned to escalate the doses still higher to establish whether further suppression of testosterone was possible. Complete suppression of testosterone synthesis should result in testosterone levels <0.7 nmol l^−1^, based upon local data, indicating that the mean value for testosterone in patients on GnRH agonist therapy is in the region of 0.7 nmol l^−1^.

In all three studies, the secondary objectives were as follows:

- to determine the safety and tolerability of abiraterone acetate in the single and multiple dose setting;
- to study the PKs of this compound; and
- to determine any other endocrine effects especially suppression of cortisol synthesis.

## INVESTIGATIONAL AGENT

Abiraterone acetate was provided by Boehringer Ingelheim as a micronised powder and prepared by the Cancer Research UK Formulation Unit (Glasgow) as 10, 50, 100 and 200 mg dry-filled capsules. The capsules were stored at room temperature.

## PATIENT POPULATION

Patients were required to be at least 18 years of age with a WHO performance status of ⩽2. No radiotherapy or hormonal therapy (with the exception of GnRHa in Study A as described above) was allowed within 6 weeks prior to study, although patients were allowed to receive concomitant bisphosphonates. Entry was further restricted to patients with haemoglobin ⩾10.0 g dl^−1^, WBC ⩾4.0 × 10^9^ l^−1^, platelet count ⩾100 × 10^9^ l^−1^, alkaline phosphatase less than twice the upper limit of normal and a urea, creatinine and bilirubin of not more than 25% above the normal range. Patients were excluded with coexistent serious nonmalignant disease and were not allowed to take concomitant steroids. All patients had stable recurrent malignancy. At the time of participation in these trials, none of the patients was considered to require any alternative therapeutic intervention for symptomatic or progressive disease.

## DOSAGE AND ADMINISTRATION

### Study A

This was a single-centre, open-label, phase one, single-dose study in which sequential cohorts of three medically or surgically castrate patients were to receive treatment at five dose levels: starting at 10 mg and increasing to 30, 100, 200 and 500 mg. Prior to study entry all patients must have had an orchidectomy or have received (and continued to receive) ongoing treatment with a GnRH agonist for at least 2 months. A confirmatory testosterone level of between 0.2 and 2.0 nmol l^−1^ was also required.

### Study B

This was a single-centre, open-label, phase one, single-dose study in which sequential cohorts of three noncastrate patients were to be treated at four dose levels: 200, 500, 650 and 800 mg. The starting dose of 200 mg was chosen when the results of study A were available. It was envisaged that a dose level would be expanded to five patients if

- one patient at any dose level experienced ⩾Grade 3 toxicity
- there was a significant suppression of serum cortisol in one patient as defined as a greater than 50% reduction in levels of cortisol, or a smaller fall associated with hypotension (systolic BP less than 90 mmHg) or persistent electrolyte disturbance.
- if the target suppression of testosterone was achieved in three patients at any one dose level.

All patients were required to have a normal testosterone level prior to study entry (i.e. ⩾9.0 nmol l^−1^) as well as normal gonadotrophin levels (Luteinising Hormone (LH) ⩽13 IU l^−1^).

### Study C

This was a three-centre, open-label, phase one, multidose study in which sequential cohorts of three noncastrate patients were to receive treatment with abiraterone daily for 12 days. All patients were required to have a normal testosterone level (⩾9.0 nmol l^−1^) as well as normal gonadotrophin levels (LH⩽13 IU l^−1^) prior to study entry.

The starting dose of 500 mg was based upon the data from the single-dose studies. It was planned that a dose level would be expanded to six patients if any patient experienced toxicity ⩾ Grade III or if there was a significant suppression of serum cortisol in one patient (defined as per Study B above). All patients were followed for 28 days for any sign of toxicity. If any patient developed symptoms suggestive of progressive disease, confirmed by a rise in PSA during the study period, they would have been offered standard treatment with a GnRH agonist.

In all studies, the capsules were administered in one oral dose at 0930 following an overnight fast. Free fluids were permitted and patients were allowed a light snack 4 h after dosing on the day of PK sampling.

## PRETREATMENT ASSESSMENT AND FOLLOW-UP INVESTIGATIONS

Prior to the first dose of therapy a complete history, physical examination and assessment of performance status was performed on all patients. Full-blood count, electrolytes and creatinine, liver function, urinanalysis, electrocardiograph and chest X-ray were obtained from all patients. The ECG was repeated 6 h after dosing. On the day of therapy heart rate, blood pressure and temperature were recorded every 4 h and then daily at the time of blood sampling. Thereafter, full-blood count, electrolytes, creatinine and liver function were re-evaluated on Days 2 and 7. Toxicity was recorded using the NCI-CTG Expanded Common Toxicity Criteria V1.

## ENDOCRINE ASSESSMENT

### Study A and B

Serum samples for endocrine analysis were obtained at 0, 2, 4, 8 and 24 h on a single day in the week prior to treatment commencement and then on Days 1, 2, 3, 4 and 7. As the duration of testosterone suppression was longer than originally anticipated, additional samples were added on Days 10, 14 and 21. The serum samples were analysed for testosterone, cortisol, 17 *α*-hydroxyprogesterone (17HP), androstenedione, LH and follicle-stimulating hormone (FSH) using commercially available kits. However, the DPC Coat-a-count kit for testosterone was sensitised using a larger volume of sample/standard and extension of the standard curve. Prior to study initiation, this was demonstrated to have no significant effect on the values of testosterone measured but to provide sensitivity to a level of 0.05 nmol l^−1^. All endocrine analyses were conducted by radioimmunoassay except for LH and FSH, which were by enzyme immunoassay.

### Study C

The schedule sampling differed slightly with samples removed at 0930 and 1730 on the day of treatment and thereafter in the morning on Days 2, 3, 4, 7, 8, 9, 10, 11, 14, 21 and 28. In Study C, a short Synacthen test was also performed prior to therapy and again around Day 11.

## PKS – ANALYTIC METHOD, ASSAYS AND SAMPLING

Sample extracts were analysed by a fully validated liquid chromatography mass spectrometry method. The instrument consisted of Wisp Model 717 autosampler including a Model 600MS system controller with a quaternary U6K LC pump. A Finnigan MAT TSQ 700 triple quadrupole mass spectrometer was used as the detection system, together with Finnigan MAT ICIS and ICL software for data capture and processing (ThermoQuest Ltd, San Jose, CA, USA). The separation of analytes was performed on a Supelcosil LC-ABZ (5 *μ*m, 250 × 4.6 mm) analytical column protected by a guard column (Supelco, Bellefonte, PA, USA). The mobile phase consisted of 570 ml of 20 mM ammonium acetate solution, 100 ml tetrahydrofuran and 1330 ml acetonitrile and was delivered at a flow rate of 1 ml min^−1^ throughout the system. Column eluant was subjected to electrospray ionisation and monitored by selected ion monitoring (SIM) of protonated pseudo-molecular ions of authentic standards of abiraterone acetate and abiraterone, and GP488 (an analogue of abiraterone used as internal standard). For SIM, the scan width was 0.25 and the total scan time was 2.99 s. Also, heated capillary temperature=250°C, spray voltage=4.5 kV, collision offset=−49.9 V and electron multiplier voltage=1200 eV.

Samples were extracted as follows: 50 *μ*l of acetonitrile and 40 *μ*l of 50 *μ*M GP488 internal standard were added to 500 *μ*l of patient plasma. After the addition of 3 ml hexane : butanol (98 : 2, v/v) and 2 min vortexing, 2 ml aliquots of the organic layer were transferred for drying *in vacuo* for 2 h. The dried residue was reconstituted by vortexing in 150 *μ*l of acetonitrile and transferred into autosampler vials. Aliquots (100 *μ*l) of these samples were injected onto the LC column. Calibration curves were obtained by plotting peak area ratios for abiraterone acetates or abiraterone to internal standard *vs* the nominal analyte concentrations using linear regression by Microsoft Excel version 5.0 (Microsoft, Redmond, WA, USA). Calibrations curves were produced at the levels of 500 and 1000 nM for abiraterone acetate and 6.25, 12.5, 25, 50, 100 and 500 nM for abiraterone. Quality controls were included at the level of 8, 40 and 400 nM for abiraterone and 500 nM for abiraterone acetate.

A comprehensive PK profile (*C*_max_, *T*_max_, *T*_1/2*α*_ *T*_1/2_ and *K*_abs_) was determined for each patient. Pharmacokinetic parameters were evaluated using WinNonLin Software® and were conducted at The Institute of Cancer Research (Sutton).

Blood was sampled for analysis of abiraterone concentration prior to drug administration and at the following times on Day 1: 30 min, 1, 2, 4, 6, 8 and 12 h. In the multidose study, samples were also drawn prior to treatment on Days 2, 3, 4, 7, 8, 9, 10, 11 and 14 with additional samples on Days 21 and 28. Samples (7 ml) were collected into vacutainer tubes (BD, Rutherford, NJ, USA) containing EDTA and immediately centrifuged to separate the plasma. At least 2 ml of plasma was transferred into polypropylene tubes (NUNC) and frozen at –20° until analysis.

## RESULTS

### Study A

A total of 16 male patients with histologically confirmed advanced adenocarcinoma of the prostate were enrolled. All patients had received previous antiandrogen therapy (flutamide or cyproterone acetate) and at time of enrolment in this study all were receiving treatment with a GnRH agonist; leuprorelin or goserelin. All patients were evaluable for safety, PK and endocrine assessments. The group had a median age of 73.5 years (Range 63–77 years) and all were performance status 0, 1.

#### Endocrine

Sequential cohorts of three patients were treated at 10, 30 and 100 mg. At these doses no consistent effect on testosterone was observed and the plasma concentrations of abiraterone were below the level of detection. Patient 4 (30 mg dose level) was observed to have noncastrate levels of testosterone during the study period despite a satisfactory screening testosterone level of 1.3 nmol l^−1^. On further questioning, it was discovered that there had been suboptimal compliance with goserelin therapy and he was deemed ineligible.

**Figure 3:**
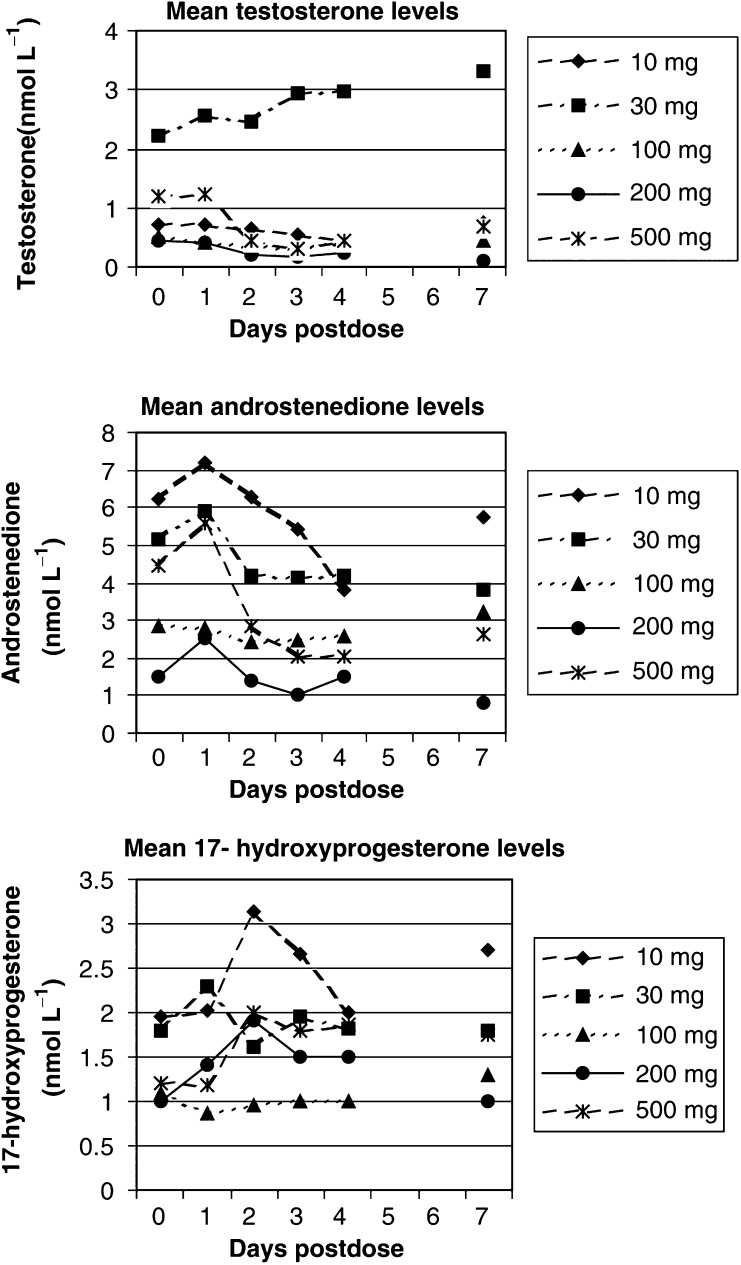
Selected mean hormone levels in castrate men receiving a single dose of abiraterone acetate.

A dose escalation to 500 mg was considered necessary as a result of the absence of a pharmacodynamic effect at doses up to 100 mg after one patient had already consented to and had received therapy at 200 mg. A 75% reduction in testosterone was observed in this patient within the first 24 h after treatment with abiraterone. In all three patients treated at 500 mg, a reduction in testosterone to the target level was seen (<0.14 nmol l^−1^ or ⩾75% reduction in baseline level testosterone ⩾0.6 mmol l^−1^). The duration of the suppression was variable. In two of the three patients, suppression was sustained from Days 2 to 5 post-therapy. Three additional patients then received treatment at 500 mg. Target testosterone suppression was seen in one of these patients. The same level of suppression was not observed in the remaining two apparently due to incorrect prior dosing with goserelin and therefore escape of testosterone levels to noncastrate levels during the study period. These results are illustrated in Figure 3

. At the 500 mg dose level, a reduction in mean androstenedione levels was parallel to the reduction in mean testosterone levels, occurring around Day 2. It was notable that there was no corresponding reduction in the levels of 17-hydroxyprogesterone. A reduction in serum cortisol levels was seen in one patient treated at 500 mg, baseline 409 nmol l^−1^, falling to 81 nmol l^−1^ Day 1. However, as this reduction was apparent at the first time point on Day 1 it was felt to be inconsistent with suppression due to abiraterone. On questioning this patient denied the concomitant use of glucocorticoids.

### Study B

Four male patients with histologically confirmed advanced adenocarcinoma of prostate were recruited. All patients had received prior antiandrogen therapy and previous therapy with a GnRH agonist but at the time of study entry had a serum testosterone of >9 nmol l^−1^. All patients were evaluable for safety, PK and endocrine assessments. The group had a median age of 71.5 years (range 60–77 years), and had performance status 0.

#### Endocrine

**Figure 4:**
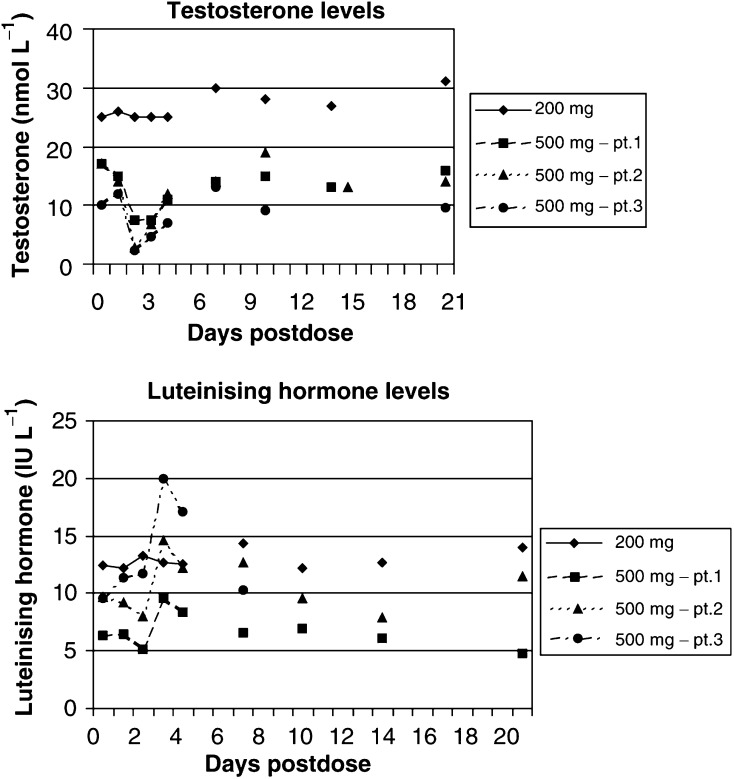
Serial hormone levels in noncastrate patients treated with a single oral dose of abiraterone acetate.

The first patient received treatment with abiraterone at 200 mg. No testosterone suppression was observed and three further patients were then treated at 500 mg. In all three patients a reduction in testosterone level of more than 50% from baseline was seen. The testosterone nadir was observed on the second day after therapy with recovery to pretreatment levels 6–9 days later. A corresponding rise in LH levels was seen (47–75%) maximal on Day 3 with recovery to pretreatment levels by Day 10 (Figure 4

). No change in cortisol level was seen.

### Study C

Six male patients with histologically confirmed advanced adenocarcinoma of the prostate were accrued. Five of the six had received prior antiandrogen therapy and the same five of six had received and completed prior therapy with a GnRH agonist. At the time of study entry, all patients had a testosterone level of >9 nmol l^−1^. The group had a median age of 68.5 years (Range 62–80 years) and had performance status 0 or 1.

#### Endocrine

**Figure 5:**
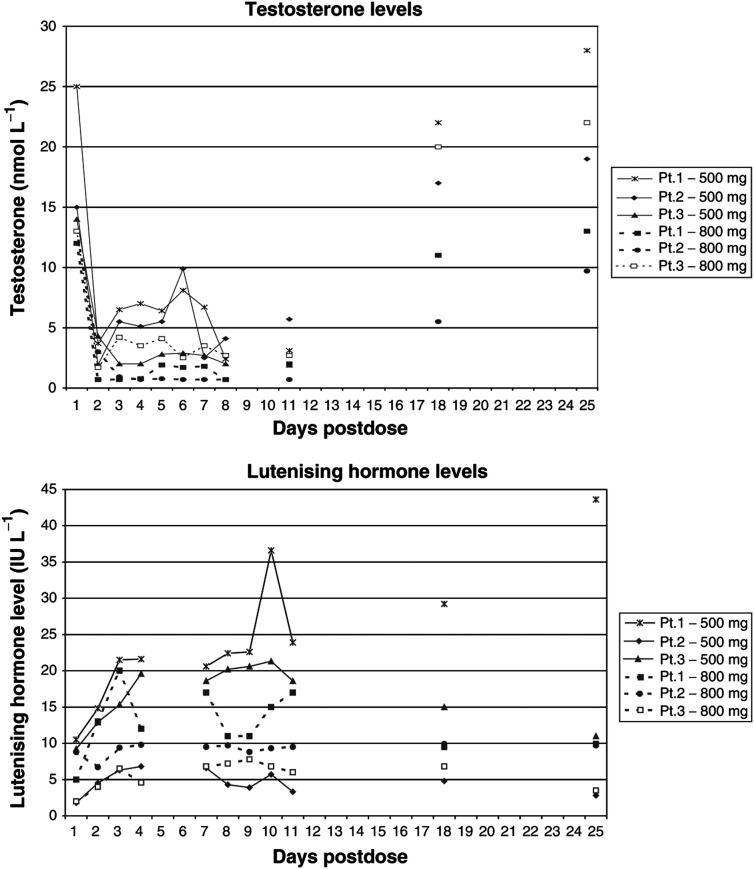
Serial hormone levels in noncastrate patients treated with multiple oral doses of abiraterone acetate.

An initial cohort of three patients received treatment at 500 mg. Although a reduction in testosterone level to ⩽2.0 nmol l^−1^ was seen in all three patients, this did not reach the target level of ⩽0.7 nmol l^−1^. The pattern of suppression was variable with maximal suppression occurring Days 1–3 and substantial suppression sustained for up to 9 days (Figure 5

). While serum cortisol levels remained within normal limits all three patients had an abnormal response to Synacthen by Day 11. The mean change in cortisol in response to Synacthen was 294.3 nmol l^−1^ (i.e. +77%) at baseline in the patients treated with 500 mg, falling to only 42 nmol l^−1^ (+10%) by Day 11. A further cohort of three patients was then treated at 800 mg to investigate whether target testosterone suppression (⩽0.7 nmol l^−1^) could be reached. In the first patient, target suppression was obtained on Day 1, sustained for 3 days and then reversed in association with rising LH (three-fold increase) from Day 3. Despite this testosterone levels remained ⩽2.0 nmol l^−1^ for the duration of treatment. In the second, target suppression was reached on Day 4, testosterone rose to 0.77 nmol l^−1^ on Day 7 but otherwise remained below the target level for the duration of treatment. In the final patient, testosterone fell to 1.7 nmol l^−1^ by Day 2 but then rose again to >2.0 nmol l^−1^ from Day 4. A concomitant two-fold rise in LH was seen from Day 3 in this patient.

The first and third patients treated at 500 mg had higher LH levels at baseline than all patients treated at 800 mg. This may have contributed to the difficulty in achieving suppression of testosterone at the lower dose level. As in those treated at 500 mg, the cortisol response to the short Synacthen test in all three patients treated at 800 mg was abnormal on Day 11. The mean change in cortisol levels in response to Synacthen was 385 nmol l^−1^ (120%) at baseline in this cohort of patients, falling to an increment of 65.3 nmol l^−1^ (23%) by Day 11. Serum cortisol levels were themselves reduced by the evening of Day 1 in three patients but all other assessments remained within normal limits. Evening cortisol falling by 60, 71 and 69%, respectively, from baseline evening cortisol in these three patients.

## PHARMACOKINETICS

The PK parameters all show considerable variability between patients and are presented in Table 1

**Table 1:** Summary of pharmacokinetic data for abiraterone acetate when given orally in single and multiple dose studies

**Patient**	**Dose (mg m^−2^)**	**AUC (μM h^−1^)**	***C*_max_ (μM)**	***T*_max_ (h)**	***T*_1/2*α*_ (h)**	***T_1/2β_* (h)**	***K*_abs_ (h)**
1	10	ND	ND	ND	ND	ND	ND
2	10	ND	0.001	2	ND	ND	ND
3	10	ND	0.018	11	ND	ND	ND
4	30	ND	ND	ND	ND	ND	ND
5	30	ND	0.004	4	ND	ND	ND
6	30	ND	0.006	1	ND	ND	ND
7	100	0.15	0.012	13	6.5	ND	6.5
8	100	0.09	0.011	0.16	0.97	26.5	0.03
9	100	0.12	0.019	3.7	2.0	28	1.8
10	200	0.39	0.061	2.8	1.59	25.8	3.87
11	500	0.25	0.063	0.8	0.28	29	0.01
12	500	1.68	0.139	3.7	1.83	21	1.8
13	500	0.73	0.06	3.6	1.73	74	1.71
14	500	0.67	0.066	4	1.43	18	1.41
15	500	0.42	0.077	2	0.29	13.4	1.00
16	500	1.38	0.167	2.7	0.05	13.3	1.15
17	200	0.23	0.037	0.69	1.49	28	0.03
18	500	1.23	0.054	1.7	0.19	14.6	0.3
19	500	1.101	0.183	1.51	0.71	14	0.7
20	500	2.08	0.30	1.41	1.59	24	0.12
21	500	3.54	0.62	1.70	1.07	23.2	0.82
22	500	0.34	0.07	2.30	0.79	12.0	0.80
23	500	NE	NE	NE	NE	NE	NE
24	800	11.66	1.19	3.02	1.32	87	1.34
25	800	2.32	0.43	1.20	0.45	19.9	0.45
26	800	2.84	0.18	3.10	1.73	26.2	1.82

Details of subjects: PT 1–16: single dose study in castrate males; PT 17–20: single dose in noncastrate males; PT 21–26: multidose study in noncastrate males. NE=not evaluated due to problems with the assay; ND=not detectable, plasma concentrations too allow to permit estimation of the pharmacokinetic behaviour of the drug; AUC=area under the concentration time curve; *C*_max_=maximum concentration, *T*_max_=time to maximum concentration; *T*_1/2*α*_=initial half-life; *T*_1/2*β*_=terminal half-life; *K*_abs_=absorption rate constant.

. The plasma concentration of abiraterone at the first three dose levels in study A was below the level of detection of the assay precluding analysis of the PK behaviour. At 100 mg, concentrations were low and the terminal half-life was unable to be determined confidently. Detectable levels were obtained at all doses greater than or equal to 200 mg. Data are not available for one patient treated at 500 mg due to an assay that failed quality control.

The mean *T*_max_ was 2.70 h (±s.d. 2.71) with a mean elimination half-life of 27.6 h (±s.d. 20.17). A range of up to 10-fold in AUC was seen for a given dose.

Within the patient groups studied, we were unable to identify any distinguishing characteristics to explain this further. The level of interpatient variability made analysis of dose-dependent PK relationships difficult. Combining the data from all three studies, while the mean AUC at each dose level increased with dose, it appeared that the association between AUC and dose is nonlinear (*R*^2^=0.34). There was no evidence of saturation of drug absorption at the dose levels studied (Table 2

**Table 2:** Summary of abiraterone clearance across all three studies

**Dose (mg)**	**Mean AUC (μM h^−1^)**
200	0.31
500	1.22
800	5.60

AUC=area under the dose/concentration curve.

and Figure 6

**Figure 6:**
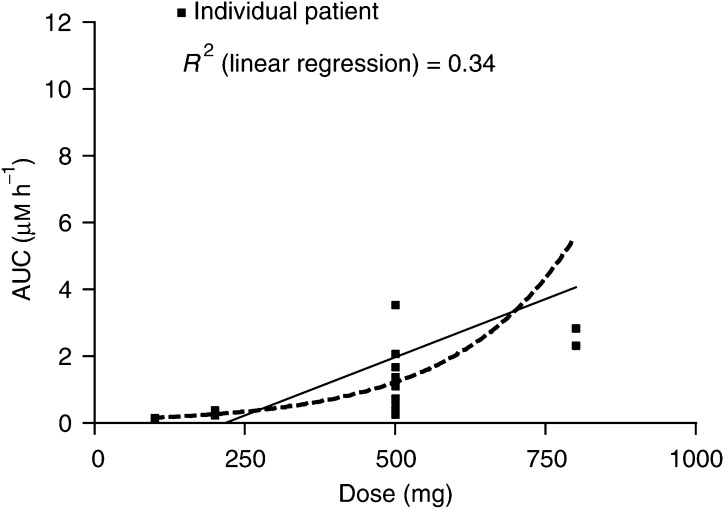
Relationship between dose and AUC for abiraterone acetate given orally for all three studies. *R*^2^=linear regression coefficient; AUC: area under the dose/concentration curve.

).

## OVERALL TOXICITY

In all three trials, abiraterone acetate was very well tolerated and no serious adverse events attributable to treatment were recorded. No haematologic or biochemical effects were observed at any dose level or schedule evaluated. No alteration in resting heart rate or blood pressure was seen. Systemic effects attributable to abiraterone were uncommon. Headache, hot flushes, a mild increase in abdominal and testicular pain, and a transient depression in mood (all grade II) were reported by individual patients but no relation to dose or schedule was apparent. There were no grade three or four toxic events.

## DISCUSSION

These are the first data to describe the systematic assessment of the endocrine effects of a specific 17*α*-hydroxylase/C_17,20_-lyase inhibitor in humans. The endocrine results that will determine the further development of abiraterone acetate are likely to be qualitatively representative of other drugs of this class.

The single dose study in castrate patients demonstrated that treatment with abiraterone acetate results in sustained suppression of the testosterone/androstenedione axis. The protracted duration of this suppression is possibly due to the irreversible nature of the drug action. In turn, therefore, one may predict that it may be possible to increase the effect with continuous dosing. This single dose study showed no effect on 17*α*-OH-progesterone production. This indicates that any inhibition of 17*α*-hydroxylation that may occur as a result of treatment with abiraterone acetate is over-ridden by compensatory mechanisms related to cortisol feedback. Despite 17*α*-hydroxylase and C_17,20_-lyase activities being contained in a single enzyme the compensated effect on 17*α*-hydroxylase activity clearly did not prevent an inhibition of C_17,20_-lyase (as evidenced by androgen suppression). Supportive evidence for this is provided by the observation that there was no significant effect on cortisol levels in these patients. Since this study was conducted in castrate patients, the data assess adrenal function as opposed to mostly testicular function.

In the single dose study of noncastrate patients, there appeared to be a steep dose–response relationship. In the patients treated at 200 mg, no effect was observed in testosterone levels and this was not because of compensation by LH (as LH levels did not alter). At 500 mg treated patients showed persistent reductions in testosterone levels. In each case the level of testosterone on Day 3 was less than 50% that of baseline, despite increased LH levels in these patients. Again there was no indication in this component study of the series that there was any effect on baseline cortisol levels despite what would appears to be a persistent block in C_17,20_-lyase activity.

From the repeat dose studies it can be seen that a dose of at least 800 mg is required to maintain testosterone suppression to target levels. In two patients treated at this dose level there was a marked rise in LH, which appeared to restrict the duration of testosterone suppression. However, in a further patient there was no compensatory LH response and testosterone levels remained very low. At 800 mg there was no effect on FSH levels apparent.

Although baseline cortisol levels remained normal, all patients treated at 500 and 800 mg in the multiple dose study developed an abnormal response to a short Synacthen test by Day 11. Some impact on adrenal reserve was predictable from the steroid synthesis pathway.

In the clinical use of both aminoglutethimide and ketoconazole, it is common practice to administer supplementary hydrocortisone and this may prove necessary with 17*α*-hydroxylase and C_17,20_-lyase inhibitors such as abiraterone acetate. However, the omission of glucocorticoid replacement when treating with aminoglutethimide and ketoconazole has been shown to be safe and effective (Eichenberger and Trachtenberg, 1988; Dowsett *et al*, 1988; Harnett *et al*, 1987; Rostom *et al*, 1982). In the light of this clinical evidence, further studies with abiraterone acetate will be required to ascertain if concomitant therapy with glucocorticoid is required on a continuous basis, at times of physiological stress, if patients become symptomatic or indeed at all.

The level of interpatient variability made analysis of dose-dependent PK relationships in these studies difficult. The majority of patients reached maximum drug concentration within 4 h of administration with an elimination half-life of approximately 29 h. However, there was up to a 10-fold variation in AUC for a given dose. This is largely accounted for by two distinct groups of outliers. Firstly, a small number of patients who absorbed the compound more quickly, reaching maximum concentration within an hour. Secondly, a group of patients in whom the elimination half-life was extremely prolonged and exceeded 70 h. Several factors may theoretically contribute to such a variation particularly with an oral compound. Patterns of absorption may be influenced by the presence of residual food in the stomach despite an overnight fast, by intrinsic interindividual differences in upper gastrointestinal pH or by interaction with other concomitant medication exerting an influence on gastric pH. Furthermore, these studies were performed using capsules containing simply loose-filled, micronised powder and we cannot exclude the possibility that this formulation might accentuate such effects. Greater interpatient consistency might be achieved with a capsule containing a more homogeneous formulation, as a melt or with excipients, to aid reproducible dissolution. Interindividual differences in body fat percentages may lead to differences in the available volume of distribution. Lastly, there may be significant interpatient differences in the rate of drug metabolism due to the effect of concomitant medication on enzyme function or pharmacogenomic characteristics.

The association between AUC and dose appeared nonlinear when results from all three studies were combined. As there were only three patients at the 800 mg level, each contributed proportionately more to the overall result. The particularly high AUC, high *C*_max_ and protracted *β*-half-life of abiraterone in patient 24 treated at 800 mg were noted; this patient may represent a true outlier to the overall relationship between AUC and dose, but additional PK information in patients receiving 800 mg would be helpful.

The degree of PK interpatient variability with abiraterone acetate is at the high end of the spectrum seen with oral anticancer compounds (DeMario and Ratain, 1998). However, as abiraterone is well tolerated and there is an appropriate and minimally invasive measure of efficacy against which to titrate the dose, this degree of variability is tolerable and should not limit clinical utility.

In addition to ketoconazole, aminoglutethimide and abiraterone, other compounds designed to inhibit general androgen production have been developed and show promise. Brodie and colleagues have described a series of novel steroidal inhibitors of androgen synthesis. The most potent of these L39, a Δ4-3-one-androstane derivative, is able to lower androgen levels effectively in animal models and inhibit tumour growth of androgen-dependent cancer cells in both *in vitro* and *in vivo* studies. It appears that this effect is not simply the result of C_17,20_-lyase and/or 5*α* reductase activity but is also mediated through a negative interaction directly with the androgen receptor (Long *et al*, 2001).

These studies demonstrate for the first time the potential utility of specific inhibition of 17*α*-hydroxylase/C_17,20_-lyase in causing reductions in testosterone levels in both castrate and noncastrate males with prostate cancer. The data indicate that reliably maintaining castrate testosterone levels in intact males in the face of increased levels of LH may require higher doses of abiraterone acetate. The present data, however, do support the potential utility of this drug in the second-line treatment of patients who have become refractory to gonadotrophin-releasing hormone agonists. In recent years, it has become routine to continue treatment with these agents in spite of disease progression, since without this, androgen stimulation may return. This being the case, a sustained further reduction in testosterone should be achievable with abiraterone acetate since the compensatory LH drive would be suppressed by the GnRH agonist. This hypothesis needs to be tested in a chronic dosing Phase I/II study in GnRH-resistant prostate cancer in the presence of continued GnRH dosing.
